# Seroprevalence of hepatitis E in adults in Brazil: a systematic review and meta-analysis

**DOI:** 10.1186/s40249-018-0514-4

**Published:** 2019-01-16

**Authors:** Fátima Mitiko Tengan, Gerusa M. Figueiredo, Arielle K. S. Nunes, Carol Manchiero, Bianca P. Dantas, Mariana C. Magri, Thamiris V. G. Prata, Marisa Nascimento, Celso C. Mazza, Edson Abdala, Antonio A. Barone, Wanderley M. Bernardo

**Affiliations:** 1 Department of Infectious and Parasitic Diseases, School of Medicine, University of São Paulo (Universidade de São Paulo - USP), São Paulo, SP Brazil; 2 Laboratory of Viral Medical Research in Hepatology (Laboratório de Investigação Médica em Hepatologia por vírus - LIM-47), Clinics Hospital, School of Medicine, USP, São Paulo, SP Brazil; 3 Instituto de Medicina Tropical da Universidade de Sao Paulo, São Paulo, SP Brazil; 4 Nursing Division, Clinics Hospital, School of Medicine, USP, São Paulo, SP Brazil; 5 School of Medicine, USP, São Paulo, SP Brazil; 6 Brazilian Medical Association (AMB), São Paulo, SP Brazil

**Keywords:** Hepatitis E, Hepatitis E virus, Brazil, Prevalence, Systematic review

## Abstract

**Background:**

Hepatitis E virus (HEV) is a member of the Hepeviridae family; it has four main genotypes and one serotype. While genotypes 1 and 2 cause epidemic hepatitis and are transmitted via water and the fecal-oral route, genotypes 3 and 4 are zoonotic. In the various seroprevalence studies of hepatitis E in Brazil, the numbers reported vary widely and are difficult to interpret. The aim of this study was to analyze existing seroprevalence studies of hepatitis E in adults in Brazil.

**Main text:**

We searched the PubMed, Latin American and Caribbean Health Sciences and Embase databases for studies published from inception to May 12, 2018 concerning infection by HEV in Brazil without time period or language restrictions. We included studies that presented data concerning hepatitis E seroprevalence in adults in Brazil, had a sample size ≥50 patients and whose method used for the detection of anti-HEV was standardized and commercialized. We also evaluated the quality of the articles using a list of criteria that totalized 9 items. Of the 20 studies ultimately analyzed, 10 (50%) were from the southeast region of Brazil, 3 (15%) were from the central-west region, 3 (15%) were from the northern region, 2 (10%) were from the northeast region and 2 (10%) were from the southern region. Regarding the quality evaluation of the studies, the mean score was 5.6 (range: 4–8). The estimated overall seroprevalence of HEV infection in the adult population was 6.0% (95% *CI*: 5.0–7.0); in subgroup analyses, we observed that the prevalence of anti-HEV antibodies in blood donors was 7.0% (95% *CI*: 5.0–8.0), whereas in the general population, it was 3.0% (95% *CI*: 2.0–4.0).

**Conclusions:**

The results of this systematic review indicate that there should be national investment in the prevention of hepatitis E virus infection in Brazil, including the implementation of improvements in basic sanitation and guidance regarding the appropriate handling of animal waste and the optimal cooking of vegetables, meat and their derivatives.

**Electronic supplementary material:**

The online version of this article (10.1186/s40249-018-0514-4) contains supplementary material, which is available to authorized users.

## Multilingual abstracts

Please see Additional file 1 for translation of the abstract into the five official working languages of the United Nations.

## Background

Hepatitis E virus (HEV) is a member of the Hepeviridae family [1], which is represented by four main genotypes and one serotype. While genotypes 1 and 2 cause epidemic hepatitis and are transmitted by water and fecal-oral route, genotypes 3 and 4 are zoonotic [2]. HEV genotypes 1 and 2 are reportedly endemic in developing countries. Genotype 1 is a major cause of acute hepatitis in Asian countries, especially in India [3], while genotype 2 is prevalent in Africa and Central America. Genotype 4 is predominant in Southeast Asia. In developed countries, HEV genotypes 1, 2 and 4 are most commonly found in patients returning from international travel; however, recent cases of autochthonous genotype 4 infections have been reported in France [4, 5]. Genotype 3 is by far the most prevalent genotype and causes autochthonous infections in developed countries. Anti-HEV antibody seroprevalence is approximately 6.0% [6] in a population-based study in the USA. In Europe, blood donor studies have shown that seroprevalence of hepatitis E ranges from 3.2% in France and 4.9% in Switzerland to 10.0% in the United Kingdom [7–9]. However, HEV is highly endemic in specific regions, such as southern France, where the seroprevalence in blood donors reaches as high as 52.5% [10].

In Brazil, the reported seroprevalence rates vary widely and are difficult to interpret. Reported seroprevalence in blood donors varies from 0.44% [11] to 10.0% [12]; rates from 2.0% [13] to 13.0% [14] have been reported in rural populations, and the prevalence ranges from 10.0% [15] to 18.8% [16] in patients with comorbid diagnosis of chronic schistosomiasis. The aim of this study was therefore to systematically evaluate the divergent seroprevalence studies of hepatitis E in adults in Brazil.

## Main text

A systematic review of published articles concerning the seroprevalence of HEV infection in adults in Brazil was carried out. Our review was conducted and reported according to the “Preferred Reporting Items for Systematic Reviews and Meta-Analyses (PRISMA) Statement,” published in 2009 [17].

### Article search strategies

We searched MEDLINE, Latin American and Caribbean Health Sciences (Literatura LatinoAmericana e do Caribe em Ciências da Saúde, Lilacs), and Embase databases for studies published from inception to May 12, 2018 regarding infection with HEV in Brazil without time period or language restrictions. In PubMed, we used the search terms [(“Hepatitis E” OR “Hepatitis E virus” OR “Hepevirus”) AND (Brazil)]. In Lilacs, we used the search terms (“Hepatite E” or “virus da Hepatite E” or “Hepevirus”). In Embase, we searched using the terms (‘hepatitis e virus’ AND ‘brazil’); more details on the search strategies are shown in Additional file 2.

We also searched for relevant studies in the references of the selected studies and in review articles on the subject. Disagreements in the identification of relevant studies were discussed by the authors until a consensus was reached. Two researchers (GMF, MCM) independently selected the eligible studies by reading the titles and abstracts, and a list of potentially relevant studies was generated. The final articles included in the review were ultimately selected (AKSN, CM) after reading the full text of each. We contacted the study authors to request data that were not present in the published study.

### Study selection

We included studies that presented data concerning hepatitis E seroprevalence in adults (age ≥ 18 years old) in Brazil, who had a sample size greater than or equal to 50 and whose method used to detect anti-HEV antibodies was standardized and commercialized. We did not include case reports, case series, review articles, comments, studies whose participants did not live in Brazil, or studies that contained the same case series presented in other publications. Regarding the latter studies, the article with the most complete data was included in our analysis.

We used the following definition for HEV infection: the presence of both IgG and IgM anti-HEV antibodies or IgG anti-HEV as measured by enzyme immunoassay using commercial kits.

### Data extraction

Two investigators (CCM, MN) collected data independently, and disagreements were resolved via discussion until reaching a consensus. The following data were collected from the articles: author name(s), year of publication, State of Brazil, population group studied, sample size, mean age, gender of the participants, number of anti-HEV antibody-positive individuals and diagnostic method for detection of anti-HEV antibodies.

### Quality assessment of the studies

To evaluate the quality of included studies, we used the Joanna Briggs Institute (JBI) Critical Appraisal Checklist for Studies Reporting Prevalence Data (Additional file 3) [18]. The purpose of this appraisal is to assess the methodological quality of a study and to determine the extent to which a study has addressed the possibility of bias in its design, conduct and analysis. The instrument is composed of 9 items; the items were scored either positive or negative, and the importance of each item was not weighted. Higher scores (positive items) correspond to higher-quality studies for our review. We considered that the studies with scores from 0 to 3, 4 to 6 and 7 to 9 presented high, moderate and low risk of bias, respectively. The quality assessment score was not used for the selection of studies for the present review. This variable (score in the quality evaluation of the study) was analyzed in meta-regression. This assessment was made independently by two researchers (BPD, TVGP), and the details of the quality assessment of the studies are shown in the Additional file 3. Disagreements during the evaluation of the quality of the studies were discussed between the researchers until a consensus was reached.

### Statistical analysis

Considering the heterogeneity expected between the studies, all of the analyses were performed using the random effects model, which contemplates the variation between studies. Heterogeneity was assessed using the *I*^2^ statistic, which describes the percentage of variation among studies that is due to heterogeneity rather than chance [19]. The following values of *I*^2^ are considered to be evidence of mild, moderate, and high heterogeneity between studies: 25–50%, 51–75% and > 75%, respectively. Low *I*^2^ values suggest that the variability between estimates is compatible with random variation.

In addition, we investigated potential sources of heterogeneity by organizing groups of studies according to potentially relevant characteristics (subgroups) and by meta-regression analysis, the objective of which was to report differences in the size of the effect of the study characteristics. We performed analyses of the following subgroups: blood donor studies, the general population (which included individuals without a diagnosis of comorbidities or risk exposure) and studies using the Abbott commercial kit for the diagnosis of anti-HEV antibodies (this commercial kit was used in 13 of the 20 selected studies). By meta-regression analysis, the following factors were examined: sample size (continuous variable), year of study publication (continuous variable), and commercial kit used for detection of anti-HEV antibodies (Abbott vs. non-Abbott) and study quality (continuous variable).

To examine the publication bias, we used tests to detect asymmetry in the funnel plot proposed by Begg and Mazumdar [20] and Egger et al. [21]. Funnel plots are scatter plots generated based on the effects we want to study, which were estimated from individual studies on the horizontal axis against some measure of study size on the vertical axis. The name ‘funnel plot’ is based on the fact that the accuracy in estimating the effect will grow as the sample size of the included studies grow. Estimates of the effects of small studies will therefore disperse substantially in the lower part of the graph, with the dispersion narrowing between larger studies. We also performed three sensitivity analyses; we excluded: (1) studies in which the score in the quality assessment of the studies was ≤5, (2) studies in which the sample size was ≤100 and (3) studies in which the sample size was ≤200.

We performed all analyses using Stata version 13 software (Stat Corp LP, Texas USA) with the commands *metan* (for random effects meta-analysis), *metareg* (for meta-regression) and *metabias* (to test asymmetry on the funnel plot).

## Results

**Fig. 1:**
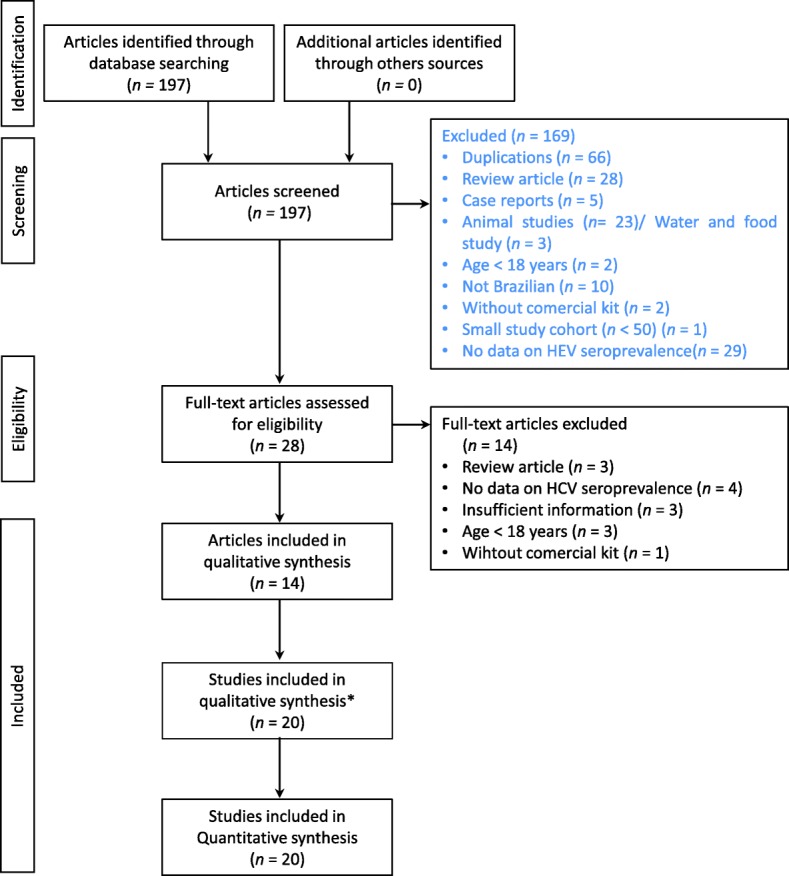
Flowchart of the identification, inclusion, and exclusion criteria of the study. * Three publications among the 14 included articles with more than one type of population, for a grand total of 20 studies

We initially identified 197 publications in the databases (MEDLINE, Lilacs and Embase), and no other sources were obtained through manual searching (Fig. 1). After the exclusion of duplicates (66), we analyzed 131 references by reading the abstracts. A hundred and three publications were subsequently excluded, leaving 28 references selected for full text reading. After reading the full text of the 28 articles, we ultimately selected 14 for final inclusion in our review. Three publications among the 14 included studies of more than one type of population, for a grand total of 20 studies (*n* = 6465). As an example, Trinta et al. [13] studied the prevalence of anti-HEV antibodies in groups of people in the general population, pregnant women, blood donors and patients undergoing hemodialysis.

Of the 20 studies concerning the prevalence of hepatitis E virus infection in Brazil, 5 (25%) were from the State of Rio de Janeiro, 5 (25%) were from the State of São Paulo, 3 were from Amazonas, (15%), 2 (10%) were from Goiás, 2 (10%) were from Bahia, 1 (5%) was from Paraná, 1 (5%) was from Santa Catarina and 1 (5%) was from Mato Grosso (Table 1). The sample sizes ranged from 65 to 996 (mean = 323, median = 263). Regarding the evaluation of the study quality, the mean score was 5.6 (median = 5.0, range = 4–8). Fourteen studies scored from 4 to 7, and 6 scored from 7 to 9.

**Table 1:** Overall characteristics of the studies selected for the review

Author	Publication year	State	Type of patient cohort	Sample size	Positive anti-HEV antibodies	Mean age	Proportion of male (%)	Commercial kit used to diagnose anti-HEV	Quality score
de Oliveira [41]	2018	Goias	Kidney transplantation	316	8	46.4	55.1	Mikrogen	8
Ferreira [42]	2018	São Paulo	HIV	93	6	48	51.6	Mikrogen	7
Bricks [43]	2018	São Paulo	Hepatitis C	585	58	53.8	53	Wantai	6
Passos-Castilho [44]	2017	São Paulo	Blood donors	500	49	38.8	49	Wantai	7
Passos-Castilho [12]	2016	Santa Catarina	Blood donors	300	30	33.2	62.7	Wantai	7
Martins [45]	2014	Goias	Waste recyclers	431	22	36.9	37.6	Mikrogen	7
Bortoliero [46]	2006	Parana	Blood donors	996	23	29.9	NA	Abbott	8
Santos [47]	2002	Rio de Janeiro	General population	530	16	NA	NA	Abbott	5
Kiesslich [11]	2002	Amazonas	Blood donors	227	1	NA	81.0	Abbott	5
Kiesslich [11]	2002	Amazonas	Pregnant women	100	0	NA	0	Abbott	5
Kiesslich [11]	2002	Amazonas	Hemodialysis	192	1	NA	60.4	Abbott	5
Trinta [13]	2001	Rio de Janeiro	Blood donors	93	4	34.6	100	Abbott	5
Trinta [13]	2001	Rio de Janeiro	Pregnant women	304	3	23.5	0	Abbott	4
Trinta [13]	2001	Rio de Janeiro	General population	145	3	31.3	52.4	Abbott	4
Trinta [13]	2001	Rio de Janeiro	Hemodialysis	65	4	65.1	47.7	Abbott	4
Gonçales [48]	2000	São Paulo	Blood donors	205	16	NA	NA	Abbott	4
Focaccia [38]	1998	São Paulo	General population	694	16	NA	NA	Abbott	6
Parana [15]	1997	Bahia	Blood donors	200	4	39	90.5	Abbott	5
Parana [15]	1997	Bahia	Hemodialysis	392	0	43	62.3	Abbott	5
Pang [49]	1995	Mato Grosso	General population	97	6	NA	NA	Genelabs Inc.	5

### Meta-analyses

**Fig. 2:**
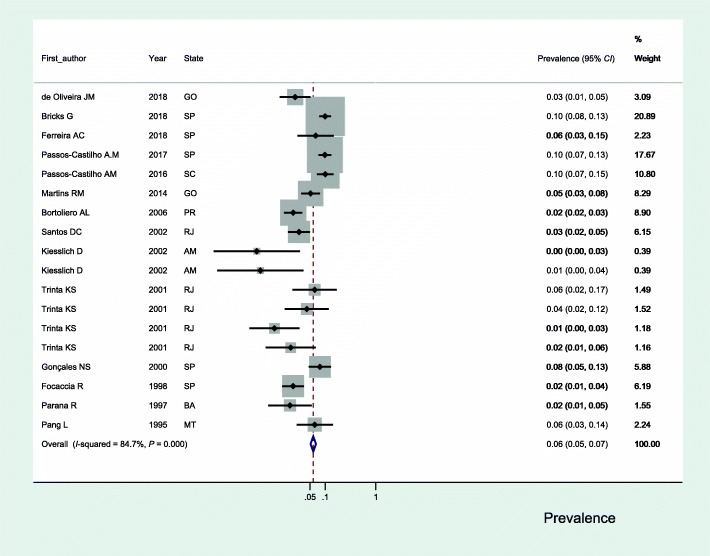
Seroprevalence of hepatitis E in adults in Brazil. GO: Goias; SP: São Paulo; SC: Santa Catarina; PR: Parana; RJ: Rio de janeiro; AM: Amazonas; BA: Bahia; MT: Mato Grosso

The estimated seroprevalence of HEV infection in the adult population of Brazil in the 20 selected studies ranged from 0.0% (95% *CI*: 0.0–3.0) to 10.0% (95% *CI*: 7.0–15.0) (Fig. 2); the heterogeneity found between the studies was substantial (*I*^2^ = 86.7%). The estimated overall prevalence was 6.0% (95% *CI*: 5.0–7.0).

**Fig. 3:**
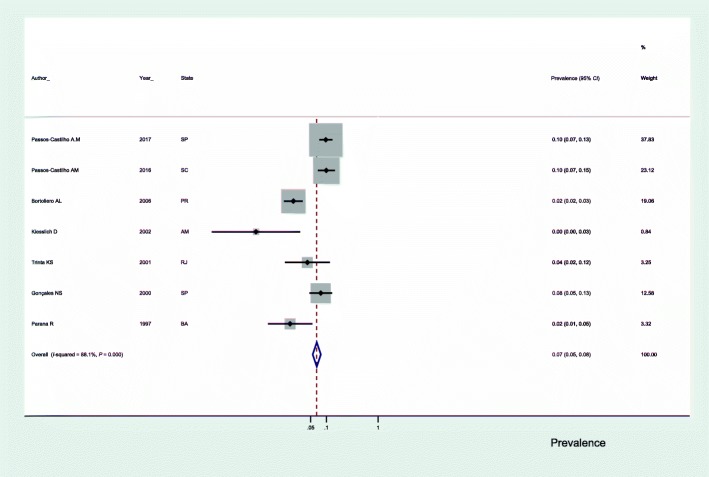
Seroprevalence of hepatitis E in blood donors in Brazil. SP: São Paulo; SC: Santa Catarina; PR: Parana; RJ: Rio de Janeiro; BA: Bahia

**Fig. 4:**
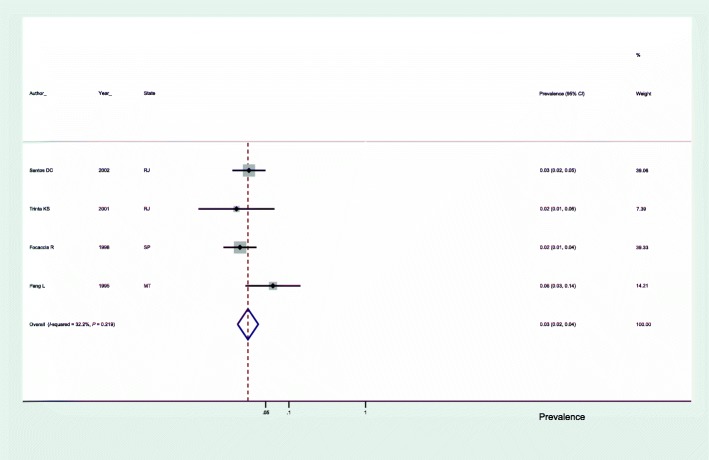
Seroprevalence of hepatitis E in the general population of Brazil. RJ: Rio de Janeiro;SP: São Paulo; MT: Mato Grosso

**Fig. 5:**
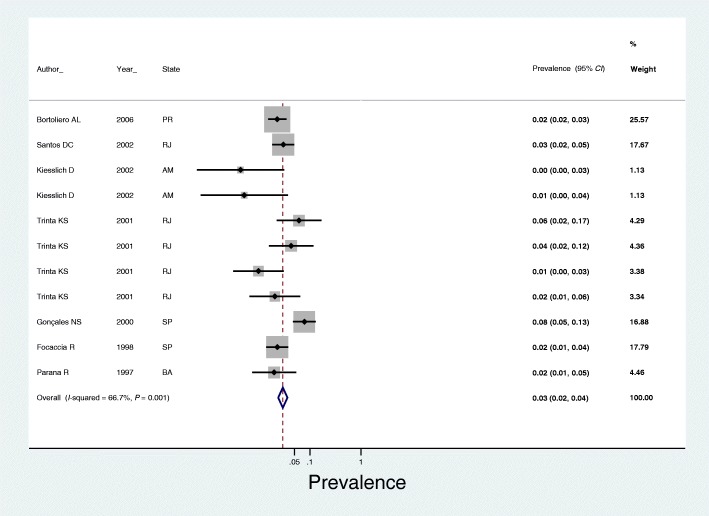
Seroprevalence of hepatitis E in studies using the Abbott commercial kit for anti-HEV antibody detection. PR: Parana; RJ: Rio de Janeiro; AM: Amazonas; SP: São Paulo; BA: Bahia

In subgroup analyses, we observed that the prevalence of anti-HEV antibodies in blood donors was 7.0% (95% *CI*: 5.0–8.0) (Fig. 3), 3.0% in the general population (95% *CI*: 2.0–4.0) (Fig. 4) and 3.0% in studies using the Abbott commercial kit for the diagnosis of anti-HEV antibodies (95% *CI*: 2.0–4.0) (Fig. 5).

Through meta-regression analysis, we evaluated the influence of the year of study publication, the sample size, the study quality for the review objectives and the type of commercial kit used for the diagnosis of anti-HEV antibodies on the seroprevalence of hepatitis E (Table 2). However, only the type of commercial kit used showed a statistically significant effect.

**Table 2:** Multivariate meta-regression analysis of the HEV seroprevalence studies in Brazil

	Meta-regression coefficient	95% *CI*	*P-*value
Publication year	0.024778	−0.0379898 − 0.0875458	0.439
Total	0.0009374	−0.0009524 − 0.0028272	0.331
Quality_score	−0.3583448	− 0.8354565 − 0.1187669	0.141
Commercial kit	1.21865	0.1469347–2.290366	0.026

The generated funnel plot appeared asymmetric (Additional file 4), and there was evidence of bias using the Egger method (*P =* 0.003) but not using the Begg method (*P =* 0.130) (Additional file 5).

Using only studies in which the quality assessment score was > 5, the overall prevalence was 7.0% (95% *CI*: 6.0–7.0) (Additional file 6). In studies with sample sizes > 100, the overall prevalence was 6.0% (95% *CI*: 5.0–7.0) (Additional file 7), and with sample sizes > 200 was 6.0% (95% *CI*: 5.0–7.0) (Additional file 8).

### Additional studies

The additional selected studies were of immunosuppressed patients (i.e., living with HIV/AIDS or having undergone kidney transplantation), patients undergoing hemodialysis, patients with hepatitis C, pregnant women and waste recyclers, as shown in Table 1. The prevalence of anti-HEV antibodies in hemodialysis patients ranged from 0 to 6.2%; in immunosuppressed people (i.e., people living with HIV/AIDS or transplanted kidneys), the variation was 2.5 to 6.5%. In patients with hepatitis C, the prevalence was 9.9%, and it was 5.1% in waste recyclers. In pregnant women, the variation was 0 to 1.0%.

## Discussion

We systematically reviewed prevalence studies of hepatitis E conducted in Brazil and identified 20 studies from 8 Brazilian states (*n* = 6465). Our review indicated that the overall seroprevalence of hepatitis E in the general adult population in Brazil was approximately 6.0% and that the heterogeneity among the studies was substantial (86.7%). Through meta-regression analysis, we observed that of the investigated factors, only the type of commercial kit used for the detection of anti-HEV antibodies showed any statistically significant influence. In subgroup analyses, we observed that the prevalence of anti-HEV antibodies was 7.0% in blood donors, 3.0% in the general population and 3.0% in the studies that used the commercial Abbott kit to identify the presence of anti-HEV antibodies. There was evidence of publication bias, but, in the sensitivity analyses, there was no significant difference in the prevalences found, indicating the robustness of the analyses performed.

Regarding countries of South America, the prevalence found in the adult population in Brazil (3.0%) is close to that reported in Uruguay (2.8%) [22] and Argentina (4.4%) [23]. In blood donors, the prevalence we determined (7.0%) is similar to that found in Chile (8.0%) [24] and greater than that found in Uruguay (1.2%) [22].

Commercial kits vary considerably in their performance, with a large variation in sensitivity and specificity. As emphasized by Hartl et al. [25], the anti-HEV IgG detected by the commercial Wantai kit generally produces higher estimates of seroprevalence than other commercial kits. In our analysis of studies that did not use the Wantai commercial kit, we did not find differences in the prevalence of anti-HEV antibodies as large as those described by Hartl et al. [25] (up to 8 times).

In the studies that used only the Abbott commercial kit, we determined a prevalence of 3.0%, and in the analysis that included the studies that used the Wantai commercial kit, we determined a prevalence of 6.0%. No definition has yet been made regarding the performance of laboratory methods capable of detecting infections that occurred in the distant past.

We believe that the difference we found in hepatitis E seroprevalence among the general population (3.0%) and blood donors (7.0%) was due to the difference in the performance of the commercial kits used for the detection of anti-HEV antibodies. Of the 2521 blood donors analyzed, 800 (32%) were analyzed with the commercial Wantai kit, with the characteristics cited above. Of the two studies (blood donors) that used the Wantai commercial kit, the seroprevalence was determined to be 9.8 and 10.0%. In the general population, the Wantai kit was not used for the detection of anti-HEV antibodies; the studies were done with the Abbott and Genelabs Inc. kits, which showed similar performances. The seroprevalence reported in the five studies of the general population ranged from 0.44 to 7.8% (Table 1). Another possibility to explain the high heterogeneity found in the studies could be the analysis of different groups, in addition to the general population and blood donors, which was done initially in our study. Due to the scarcity of studies in each of these groups (hemodialysis, immunosuppressed, hepatitis C, pregnant women and waste recyclers), it was not possible to perform the analysis by group of participants.

Although we have found evidence of publication bias, the sensitivity analyses appear to have shown, in our opinion, the robustness of the data obtained through the meta-analysis of the 20 selected studies. The overall prevalence confidence interval of the 20 studies (95% *CI*: 5.0–7.0) overlaps with the prevalence observed in the sensitivity analyses, which sought to determine whether the exclusion of studies with smaller sample sizes or lower scores in the quality assessment of the studies would reduce the prevalence rate of anti-HEV antibodies.

Based on studies of individuals who have undergone kidney transplantation [26] and patients with previous diagnosis of non-A, non-B hepatitis [27], there are indications that hepatitis E virus genotype 3 is circulating among humans in Brazil.

HEV genotypes 3 and 4 are recognized for infecting humans and animals, in contrast to genotypes 1 and 2; pigs, deer and boars have been identified as reservoirs of infection [28, 29]. In a study conducted in northeast China, the authors observed that the prevalence of anti-HEV antibodies was 31.6, 28.6 and 21.1% in individuals with frequent, infrequent and very rare contact with pigs [30]. There is also evidence that ingestion of raw or medium-raw meat and pig offal can transmit HEV [31]. In addition, HEV RNA has been found in commercially-available swine livers and products derived from swine containing raw liver tissue [32], in mussels [33], in oysters [34] and in seafood [35]. It has also been found in strawberries in Canada [36] and in the supply chain of vegetables for salads in Europe [37]. It is likely that the presence of HEV in mollusks and vegetables is due to their contamination with water with animal sewage.

Our study has certain limitations. Studies of the prevalence of hepatitis E in the general population, as we have defined it, are not available in several Brazilian states. Except for the study by Focaccia et al. [38], we did not include another population-based study. We think it would be inconvenient to perform a more in-depth analysis of the study groups, such as those of the immunosuppressed individuals, the hemodialysis patients and the waste recyclers because the numbers of these individuals are too small to allow further inferences.

The results of this review indicate that preventive measures should be undertaken to avoid infection with the hepatitis E virus. Efficient inactivation of HEV in infected food products derived from pig livers is reportedly only achieved after a cooking time of at least 20 min at an internal temperature of 71 °C [39, 40]. Appropriate hygienic measures must be taken when handling raw meat. In addition, pig waste must be disposed of properly, and the use of pig manure as a soil fertilizer should be regulated to reduce the risk of HEV contamination of plants and surface water.

## Conclusions

The estimated overall seroprevalence of hepatitis E in the Brazilian adult population was 6.0% and in subgroup analyses was 7.0% in blood donors and 3.0% in the general population, showing that HEV infection is not rare in Brazil. It is a matter of concern and alert the need for investment in the prevention of infection with HEV in Brazil that should include the improvement in basic sanitation by the government and the development and provision of guidance for the appropriate handling of waste animal and safe cooking of vegetables, meat and their derivatives. Further, it became clear that more studies are needed in different population groups and in other countries to completely understand the magnitude of the epidemiology of hepatitis E infection in Brazil.

## Additional files

Additional file 1: Multilingual abstracts in the five official working languages of the United Nations. (PDF 748 kb)

Additional file 2: Search strategies. Search strategies used in registry databases. (DOCX 14 kb)

Additional file 3: A Instrument for assessment of the quality of the studies. B Assessment of the quality of the studies. Quality assessment of the selected studies. (DOCX 31 kb)

Additional file 4: Funnel plot analysis proposed by Begg and Mazumdar [19] to study publication bias. Funnel chart of selected studies. (PDF 47 kb)

Additional file 5: Tests for Publication Bias. Tests for Publication Bias. (DOCX 11 kb)

Additional file 6: Seroprevalence of Hepatitis E in studies with a quality evaluation score > 5. (PDF 26 kb)

Additional file 7: Seroprevalence of Hepatitis E in studies with a sample size > 100. (PDF 26 kb)

Additional file 8: Seroprevalence of hepatitis E in studies with a sample size > 200. (PDF 26 kb)

## Abbreviations

HEV: Hepatitis E virus
JBI: Joanna Briggs Institute
Lilacs: Literatura Latino Americana e do Caribe em Ciências da Saúde
PRISMA: Preferred Reporting Items for Systematic Reviews and Meta-Analyses
