# Supplementary material for: Seroprevalence of hepatitis E in adults in Brazil: a systematic review and meta-analysis
Source: Infect Dis Poverty. 2019 Jan 16;8:3. doi: 10.1186/s40249-018-0514-4 (PMC6334402; doi:10.1186/s40249-018-0514-4)
Supplement: Supplementary file 1 — Multilingual abstracts in the five official working languages of the United Nations. (PDF 748 kb) [file 40249_2018_514_MOESM1_ESM.pdf]

Translation of the abstract into the five official working languages of the United Nations

الانتشار المصلي لالتهاب الكبد E لدى البالغين في البرازيل: مراجعة منهجية وتحليل تلوي

Mariana C. ،Bianca P. Dantas ،Carol Manchiero ،Arielle KS Nunes ،Gerusa M. Figueiredo ،Fátima Mitiko Tengan
Wanderley M. ،Antonio A. Barone ،Edson Abdala ،Celso C. Mazza ،Marisa Nascimento ،Thamiris VG Prata ،Magri
Bernardo

الملخص

خلفية: فيروس التهاب الكبد الوبائي (HEV) هو عضو في الأسرة هيبيفيريداي، وله أربعة تراكيب وراثية الرئيسية ونمط المصلي واحد. في حين أن التراكيب الوراثية 1 و 2 تسبب الالتهاب الكبدي الوبائي وتنتقل عن طريق المياه والطريق البرازي الفموي ، فإن الأنماط الجينية 3 و 4 هي حيوانية. في مختلف الدراسات حول الانتشار المصلي للالتهاب الكبدي "E" في البرازيل ، تتفاوت الأعداد المذكورة على نطاق واسع ويصعب تفسيرها. كان الهدف من هذه الدراسة هو تحليل دراسات الانتشار المصلي الموجودة من التهاب الكبد E لدى البالغين في البرازيل.

النص الرئيسي: بحثنا في قواعد بيانات PubMed، والعلوم الصحية في أمريكا اللاتينية ومنطقة البحر الكاريبي وقواعد البيانات Embase للدراسات المنشورة منذ البداية إلى 12 مايو 2018 فيما يتعلق بالإصابة بعدوى (HEV) في البرازيل دون فترة زمنية أو قيود اللغة. قمنا بتضمين الدراسات التي قدمت بيانات تتعلق بالانتقال المصلي للالتهاب الكبدي E لدى البالغين في البرازيل ، وكان حجم العينة ≤ 50 مريضا ، وطريقة استخدامها للكشف عن HEV-anti تم توحيدها وتسويقها. قمنا أيضًا بتقييم جودة المقالات باستخدام قائمة معايير مجموعها 9 عناصر. من الدراسات 20 التي جرى تحليلها في نهاية المطاف، كانت 10 (50 ٪) من منطقة جنوب شرق البرازيل و3 (15 في المائة)من المنطقة الغربية الوسطى، و3 (15 في المائة) من المنطقة الشمالية، 2 (10%) من منطقة شمال شرق وكانت 2 (10 ٪) من منطقة الجنوب. فيما يتعلق بتقييم جودة الدراسات ، كانت النتيجة المتوسطة 5.6 (المدى: 4–8). بلغ معدل الانتشار المصلي الإجمالي للعدوى HEV في السكان البالغين 6.0 ٪ (95 ٪ CI: 5.0–7.0) ؛ في تحليلات المجموعات الفرعية ، لاحظنا أن انتشار الأجسام المضادة ل HEV في المتبرعين بالدم كان 7.0 ٪ (95 ٪ CI: 5.0-8.0) ، في حين أنه في عموم السكان ، كان 3.0٪ (95٪ CI: 2.0–4.0).

الاستنتاجات تشير نتائج هذه المراجعة المنهجية إلى أنه يجب أن يكون هناك الاستثمار الوطني في الوقاية من العدوى بفيروس التهاب الكبد الوبائي (HEV) في البرازيل، بما في ذلك تنفيذ تحسينات في المرافق الصحية الأساسية والتوجيه فيما يتعلق بالتعامل المناسب مع النفايات الحيوانية والطهي الأمثل من الخضروات واللحوم ومشتقاتها

Translated from English version into Arabic by Kowthar Alasady, proofread by Heba Kandel, through

TRANSLATORS
WITHOUT BORDERS

巴西成人戊型肝炎的血清阳性率：一项系统综述和荟萃分析

Fátima Mitiko Tengan, Gerusa M. Figueiredo, Arielle K. S. Nunes, Carol Manchiero, Bianca P. Dantas, Mariana C. Magri, Thamiris V. G. Prata, Marisa Nascimento, Celso C. Mazza, Edson Abdala, Antonio A. Barone, Wanderley M. Bernardo

摘要

引言：戊型肝炎病毒(HEV)隶属于戊型肝炎病毒科（Hepeviridae），包括 4 种主要的基因型和 1 种血清型。基因 1 型和 2 型可引起流行性肝炎，通过水和粪口途径传播；基因 3 型和 4 型是人人兽共患型。在巴西的各种戊型肝炎血清阳性率研究中，报道的数据差异很大，且难以解释。本研究旨在分析巴西成人戊型肝炎的现有血清阳性率情况。

主要内容：我们检索了PubMed、拉丁美洲和加勒比卫生科学和Embase数据库，囊括了截止到 2018年5月12日发表的巴西HEV感染研究(无时间和语言限制)。纳入研究的条件为：含有关于巴西成年人HEV血清阳性率数据，样本量≥ 50，且使用标准化和商业化戊型肝炎抗体检测。我们还使用一个总共包含9个项目的标准列表来评估文章质量。在最终分析的20项研究中，包含来自巴西东南部10项(50%)，中西部3项（15%），北部3项（15%），东北部2项（10%），南部2项（10%）。关于研究的质量评价，平均分数为5.6分(范围：4−8)。估计成人HEV总血清阳性率为6.0%（95% *CI*：5.0−7.0）；在亚组分析中，我们发现抗献血者的HEV抗体阳性率为7.0%（95% *CI*：5.0−8.0），而在一般人群中为3.0%（95% *CI*：2.0−4.0）。
结论：本系统综述的研究结果表明，巴西应在预防戊型肝炎病毒感染方面进行国家投入，包括改善基本卫生条件，适当处理动物粪便，以及最佳烹调蔬菜、肉类及其衍生物等方面进行指导。

Translated from English version into Chinese by Xin-Yu Feng, edited by Pin Yang

CHINESE CENTER FOR DISEASE CONTROL AND PREVENTION
NATIONAL INSTITUTE OF PARASITIC DISEASES

La séroprévalence de l'hépatite E chez les adultes au Brésil: examen systématique et méta-analyse

Fátima Mitiko Tengan, Gerusa M. Figueiredo, Arielle K. S. Nunes, Carol Manchiero, Bianca P. Dantas, Mariana C. Magri, Thamiris V. G. Prata, Marisa Nascimento, Celso C. Mazza, Edson Abdala, Antonio A. Barone, Wanderley M. Bernardo

Résumé

Contexte: Le virus de l’hépatite E (VEH) est un membre de la famille des Hepeviridae; il compte quatre génotypes principaux et un sérotype. Alors que les génotypes 1 et 2 entraînent une hépatite épidémique et sont transmis par l’eau et la voie féco-orale, les génotypes 3 et 4 sont des zoonoses. Dans les différentes études portant sur la séroprévalence de l’hépatite E au Brésil, les données recueillies varient considérablement et sont difficiles à interpréter. La présente étude a pour objectif d’analyser les études réalisées au Brésil sur la séroprévalence de l’hépatite E chez les adultes.
Texte principal: Nous avons effectué une recherche sur les études publiées concernant les infections par le VHE au Brésil, sans restriction de durée ou de langues, dans les bases de données de PubMed, du Latin American and Caribbean Health Sciences et d’Embase, depuis la création de ces dernières jusqu’au 12 mai 2018. Nous avons retenu les études présentant des données sur la séroprévalence de l'hépatite E chez les adultes au Brésil, utilisant un échantillon de taille ≥ 50 patients et employant une méthode de détection des anti-VHE normalisée et commercialisée. Nous avons également évalué la qualité des articles en fonction d’une liste de critères comptant 9 éléments. Sur les 20 études finalement analysées, 10 (50 %) provenaient du sud-est du Brésil, 3 (15 %) du centre-ouest, 3 (15 %) du nord, 2 (10 %) du nord-est et 2 (10%) du sud. En ce qui concerne l’évaluation de la qualité des études, le score moyen était de 5,6 (intervalle: 4-8). La séroprévalence globale de l’infection par le VHE dans la population adulte était estimée à 6,0 % (95 % *IC*: 5,0−7,0); dans des analyses de sous-groupes, nous avons observé que la prévalence des anticorps anti-VHE chez les donneurs de sang était de 7,0 % (95 % *IC*: 5,0−8,0), alors que dans la population générale, elle était de 3,0 % (95 % *IC*: 2,0−4,0).
Conclusions: Les résultats du présent examen systématique indiquent que le Brésil doit investir dans la prévention de l'infection par le virus de l’hépatite E, notamment en œuvrant à l’amélioration de l’assainissement de base et en émettant des recommandations sur la bonne manipulation des déchets d’origine animale et sur les bonnes méthodes de cuisson des légumes, de la viande et des produits qui en dérivent.

Translated from English version into French by Cendrine Strevens, proofread by Suzanne Assenat, through

Серопревалентность гепатита Е у взрослых в Бразилии: систематический обзор и метаанализ

Фатима Митико Тенган (Fátima Mitiko Tengan), Джеруса М. Фигейредо (Gerusa M. Figueiredo), Ариэль КС Нунес (Arielle KS Nunes), Кэрол Маншьеро (Carol Manchiero), Бьянка П. Дантас (Bianca P. Dantas), Мариана С. Магри (Mariana C. Magri), Тамирис ВГ Прата (Thamiris VG Prata), Мариса Нашименто (Marisa Nascimento), Сельсо С. Мазза (Celso C. Mazza), Эдсон Абдала (Edson Abdala), Антонио А. Бароне (Antonio A. Barone), Уондерли М. Бернардо (Wanderley M. Bernardo)

Аннотация

Справочная информация: Вирус гепатита Е (HEV) принадлежит к семейству Hepeviridae; в его состав входят четыре основных генотипа и один серотип. В то время как генотипы 1 и 2 вызывают эпидемический гепатит и передаются через воду, а также фекально-оральным путем, генотипы 3 и 4 являются зоонозными. В различных исследованиях в области серопревалентности гепатита Е в Бразилии указанные цифры широко варьируются и с трудом поддаются интерпретации. Целью данного исследования ставился анализ существующих исследований серопревалентности гепатита Е у взрослых в Бразилии.

Основной текст:В базах данных PubMed, Embase, а также в исследовательских базах по вопросам здравоохранения Латинской Америки и Карибского бассейна за период с момента возникновения и до 12 мая 2018 года был произведён поиск информации об исследованиях, касающихся инфекции HEV в Бразилии без учёта временных или языковых ограничений. Мы включили исследования, в которых были представлены данные о серпревалентности гепатита Е у взрослых в Бразилии, размер образца для исследования составлял ≥ 50 больных, а метод, использованный для обнаружения анти-HEV, был стандартизирован и коммерциализирован. Используя список критериев, который включал 9 позиций, мы также произвели оценку качества статей. Из проанализированных 20 исследований 10 (50%) относились к юго-восточному региону Бразилии, 3 (15%) были из центрально-западного региона, 3 (15%) — из северного региона, 2 (10%) — из северо-восточного региона и 2 (10%) — из южного региона. Что касается оценки качества исследований, средний балл составил 5,6 (диапазон: 4-8). Согласно произведённым оценкам, общая серопревалентность инфекции HEV среди взрослого населения составила 6,0% (95% *CI*: 5,0−7,0); в анализах подгрупп распространённость антител анти-HEV в крови доноров наблюдалась на уровне 7,0% (95% *CI*: 5,0−8,0), тогда как у населения в целом этот показатель составил 3,0% (95% *CI*: 2,0−4,0).

Выводы: Результаты данного систематического обзора показывают, что государство должно инвестировать в профилактику заражения вирусом гепатита Е в Бразилии, в том числе внедрение усовершенствований в базовую санитарно-гигиеническую деятельность, а также руководство относительно надлежащей обработки отходов животноводства и оптимального приготовления овощей, мяса и их производных.

Translated from English version into Russian by Liudmila Tomanek, proofread by Olga Madiar, through

Seroprevalencia de la hepatitis E en adultos en Brasil: una revisión sistemática y metaanálisis

Fátima Mitiko Tengan, Gerusa M. Figueiredo, Arielle K. S. Nunes, Carol Manchiero, Bianca P. Dantas, Mariana C. Magri, Thamiris V. G. Prata, Marisa Nascimento, Celso C. Mazza, Edson Abdala, Antonio A. Barone, Wanderley M. Bernardo

Resumen

Introducción: El virus de la hepatitis E (VHE) es un miembro de la familia Hepeviridae; tiene cuatro genotipos principales y un serotipo. Mientras que los genotipos 1 y 2 causan hepatitis epidémica y se transmiten a través del agua y la vía fecal-oral, los genotipos 3 y 4 son zoonóticos. En los diversos estudios de seroprevalencia de hepatitis E en Brasil, los números informados varían mucho y son difíciles de interpretar. El objetivo de este estudio fue analizar los estudios existentes de seroprevalencia de hepatitis E en adultos en Brasil.

Texto principal: Se realizaron búsquedas en las bases de datos de PubMed, Ciencias de la Salud de América Latina y el Caribe y Embase de estudios publicados desde el inicio hasta el 12 de mayo de 2018 sobre la infección por el VHE en Brasil sin restricciones de idioma o período de tiempo. Se incluyeron estudios que presentaban datos sobre la seroprevalencia de hepatitis E en adultos en Brasil, tenían un tamaño de muestra de ≥ 50 pacientes y cuyo método utilizado para la detección de anti-VHE estaba estandarizado y comercializado. También se evaluó la calidad de los artículos utilizando una lista de criterios con un total de 9 ítems. De los 20 estudios que se analizaron en última instancia, 10 (50%) eran de la región sudeste de Brasil, 3 (15%) de la región centro-oeste, 3 (15%) de la región norte, 2 (10%) de la región noreste y 2 (10%) de la región sur. Con respecto a la evaluación de la calidad de los estudios, la puntuación media fue de 5.6 (gama: 4−8). La seroprevalencia global estimada de infección por VHE en la población adulta era del 6.0 % (95 % *CI*: 5.0−7.0); en los análisis de subgrupos, se observó que la prevalencia de anticuerpos anti-VHE en donantes de sangre era de 7.0 % (95 % *CI*: 5.0−8.0), mientras que en la población general era de 3.0 % (95 % *CI*: 2.0−4.0).

Conclusiones: Los resultados de esta revisión sistemática indican que debería haber inversión nacional en la prevención de la infección por el virus de la hepatitis E en Brasil, incluida la implementación de mejoras en el saneamiento básico y la orientación sobre el manejo adecuado de los desechos animales y la cocción óptima de verduras, carne y sus productos derivados.

Translated from English version into Spanish by Eugenia Cagni, proofread by Kate Pattison, through
