# Supplementary material for: Seroprevalence of hepatitis E in adults in Brazil: a systematic review and meta-analysis
Source: Infect Dis Poverty. 2019 Jan 16;8:3. doi: 10.1186/s40249-018-0514-4 (PMC6334402; doi:10.1186/s40249-018-0514-4)
Supplement: Supplementary file 2 — Search strategies. Search strategies used in registry databases. (DOCX 14 kb) [file 40249_2018_514_MOESM2_ESM.docx]

**Additional file 2: Search strategies**

**PubMed=62**

Words or Terms : ("Hepatitis E"[Mesh] OR "Hepatitis E virus"[Mesh] OR "Hepevirus"[Mesh] OR “Hepatitis E” [word] OR “hepatitis e virus” [word] OR “Hepevirus” [word]) AND (brazil)

**Lilacs=20**

Words or Terms: (Hepatite E OR virus da hepatite E) [Palavras] and brasil

**Embase: 115**

Words or Terms: ‘hepatitis e virus’ AND ‘brazil’
