# Supplementary material for: Seroprevalence of hepatitis E in adults in Brazil: a systematic review and meta-analysis
Source: Infect Dis Poverty. 2019 Jan 16;8:3. doi: 10.1186/s40249-018-0514-4 (PMC6334402; doi:10.1186/s40249-018-0514-4)
Supplement: Supplementary file 3 — A Instrument for assessment of the quality of the studies. B Assessment of the quality of the studies. Quality assessment of the selected studies. (DOCX 31 kb) [file 40249_2018_514_MOESM3_ESM.docx]

**Additional file 3**

1. **Instrument for assessment of the quality of the studies**

**JBI Critical Appraisal Checklist for Studies Reporting Prevalence Data**

Reviewer Date

Author Year Record Number

|  | Yes | No | Unclear | Not applicable |
| --- | --- | --- | --- | --- |
| 1. Was the sample frame appropriate to address the target population? | □ | □ | □ | □ |
| 1. Were study participants sampled in an appropriate way? | □ | □ | □ | □ |
| 1. Was the sample size adequate? | □ | □ | □ | □ |
| 1. Were the study subjects and the setting described in detail? | □ | □ | □ | □ |
| 1. Was the data analysis conducted with sufficient coverage of the identified sample? | □ | □ | □ | □ |
| 1. Were valid methods used for the identification of the condition? | □ | □ | □ | □ |
| 1. Was the condition measured in a standard, reliable way for all participants? | □ | □ | □ | □ |
| 1. Was there appropriate statistical analysis? | □ | □ | □ | □ |
| 1. Was the response rate adequate, and if not, was the low response rate managed appropriately? | □ | □ | □ | □ |

Overall appraisal: Include □ Exclude □ Seek further info □

Comments (Including reason for exclusion)

1. **Assessment of the quality of the studies**

| **AUTHOR (year)** | **TOTAL SCORE** |
| --- | --- |
| De Oliveira (2018) | 8 |
| Bricks (2018) | 7 |
| Ferreira (2018) | 6 |
| Passos-Castilho (2017) | 7 |
| Passos-Castilho (2016) | 7 |
| Martins (2014) | 7 |
| Bortoliero (2006) | 8 |
| Santos (2012) | 5 |
| Kiesslich (2002) | 5 |
| Trinta (2001) | 5 |
| Gonçales (2000) | 4 |
| Focaccia (1998) | 6 |
| Parana (1997) | 5 |
| Pang (1995) | 5 |
