# Supplementary figures and images for: Seroprevalence of hepatitis E in adults in Brazil: a systematic review and meta-analysis
Source: Infect Dis Poverty. 2019 Jan 16;8:3. doi: 10.1186/s40249-018-0514-4 (PMC6334402; doi:10.1186/s40249-018-0514-4)

Begg's funnel plot with pseudo 95% confidence interval

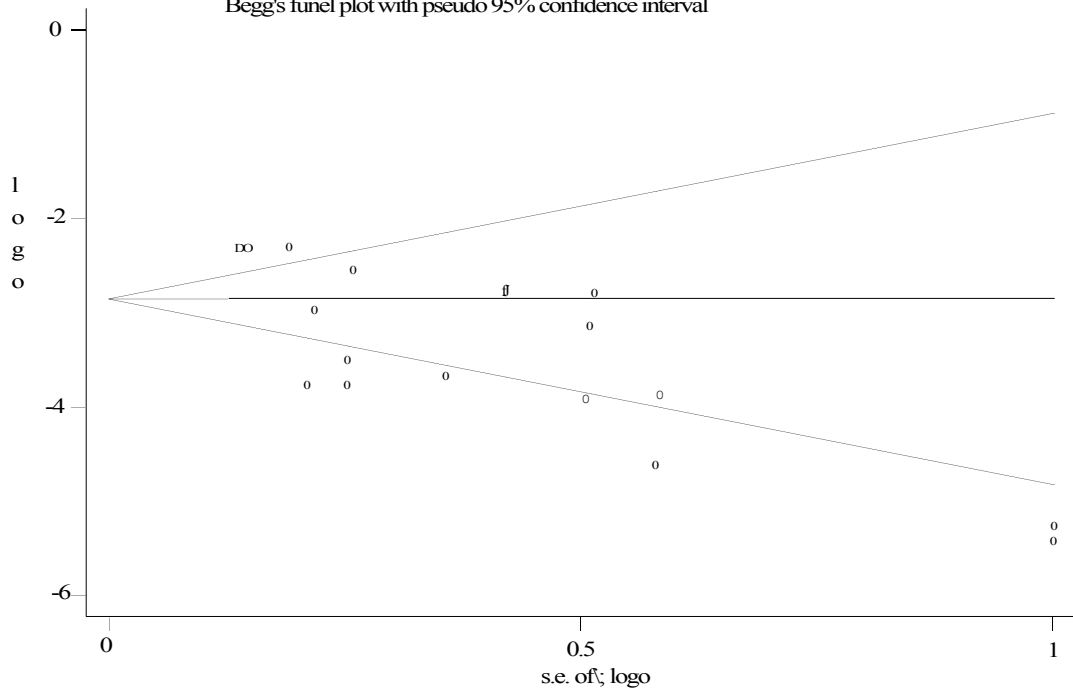

Supplement: Supplementary file 4 — Funnel plot analysis proposed by Begg and Mazumdar [19] to study publication bias. Funnel chart of selected studies. (PDF 47 kb) [file 40249_2018_514_MOESM4_ESM.pdf]

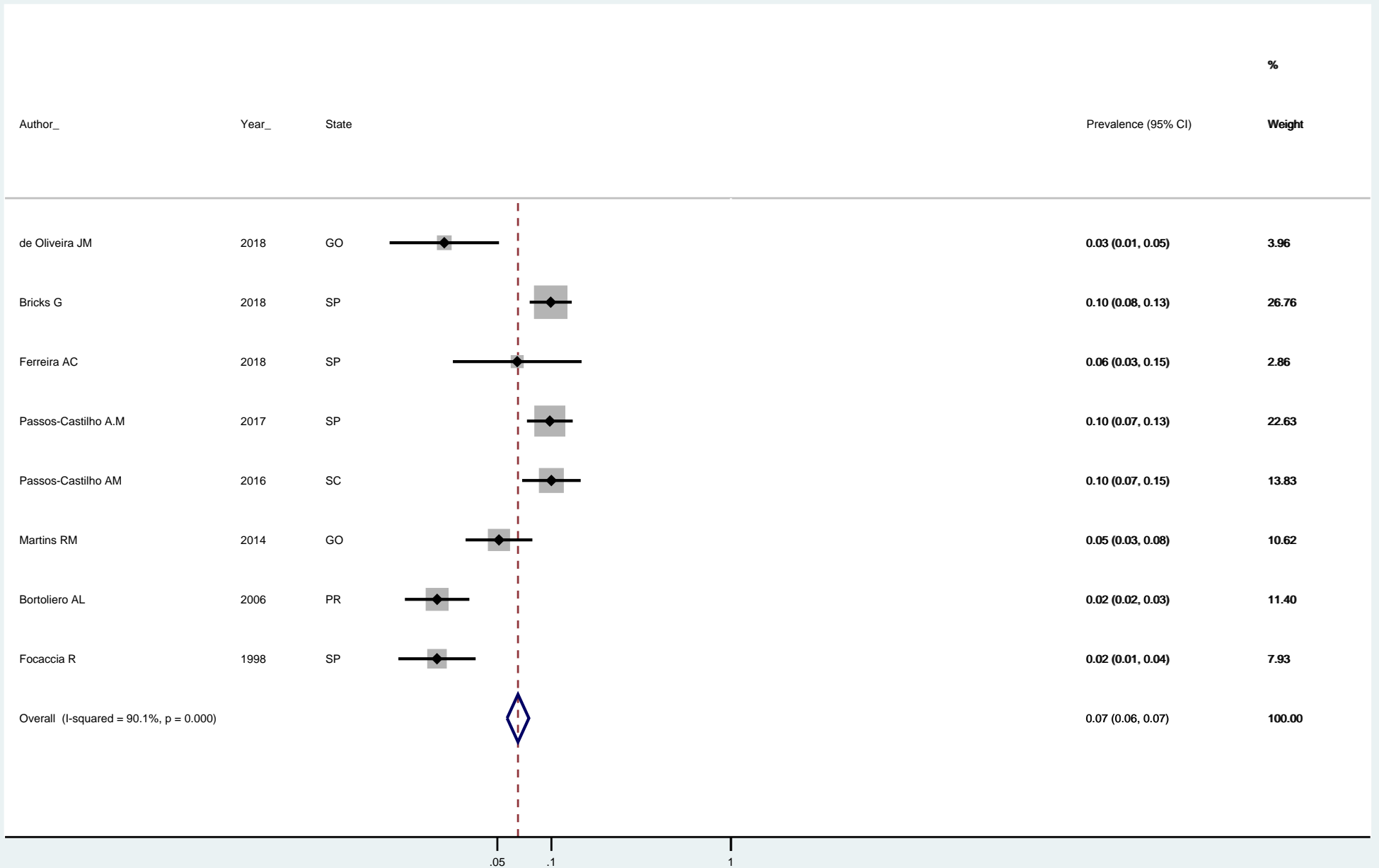

Prevalence

Supplement: Supplementary file 6 — Seroprevalence of Hepatitis E in studies with a quality evaluation score > 5. (PDF 26 kb) [file 40249_2018_514_MOESM6_ESM.pdf]

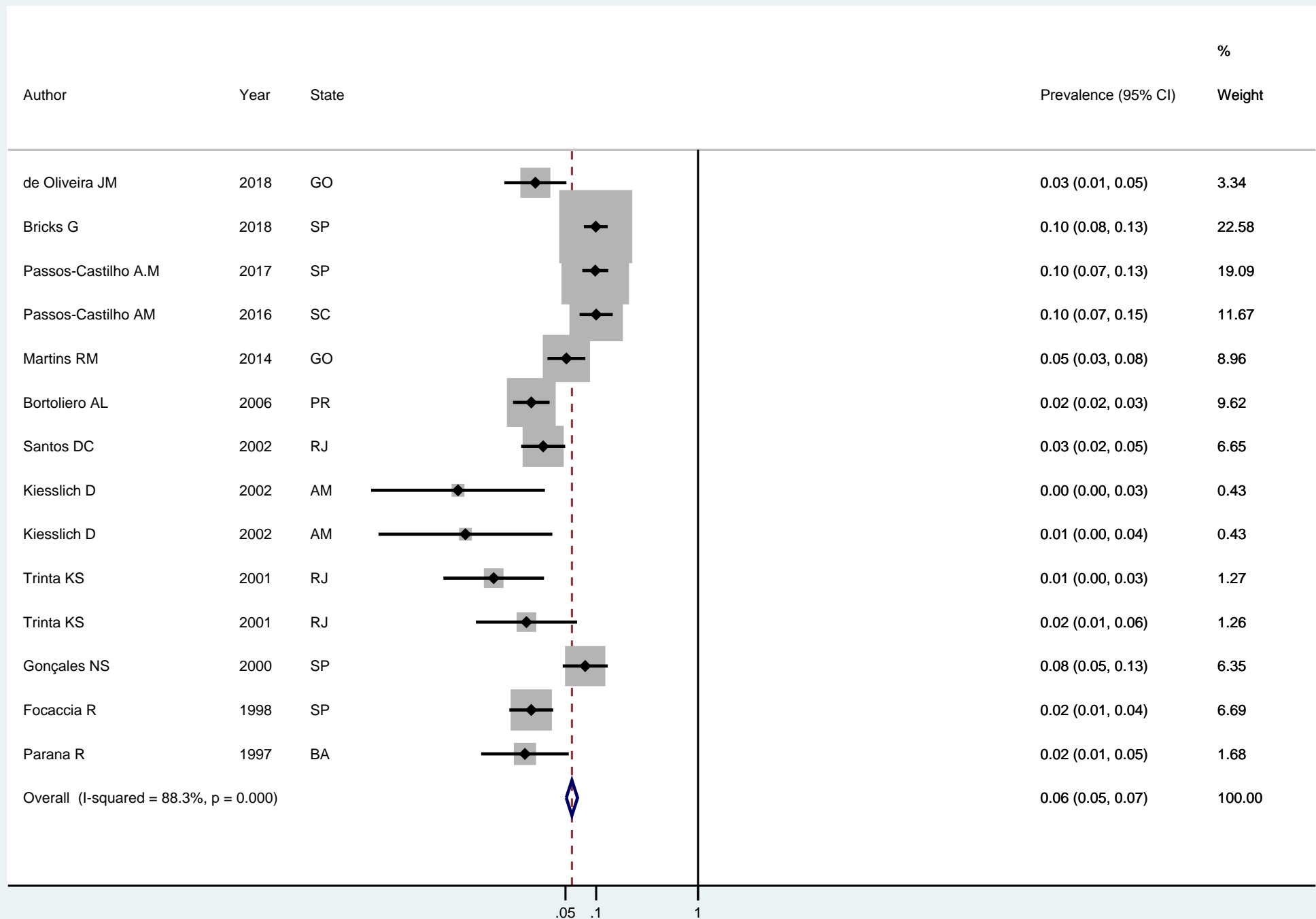

Supplement: Supplementary file 7 — Seroprevalence of Hepatitis E in studies with a sample size > 100. (PDF 26 kb) [file 40249_2018_514_MOESM7_ESM.pdf]

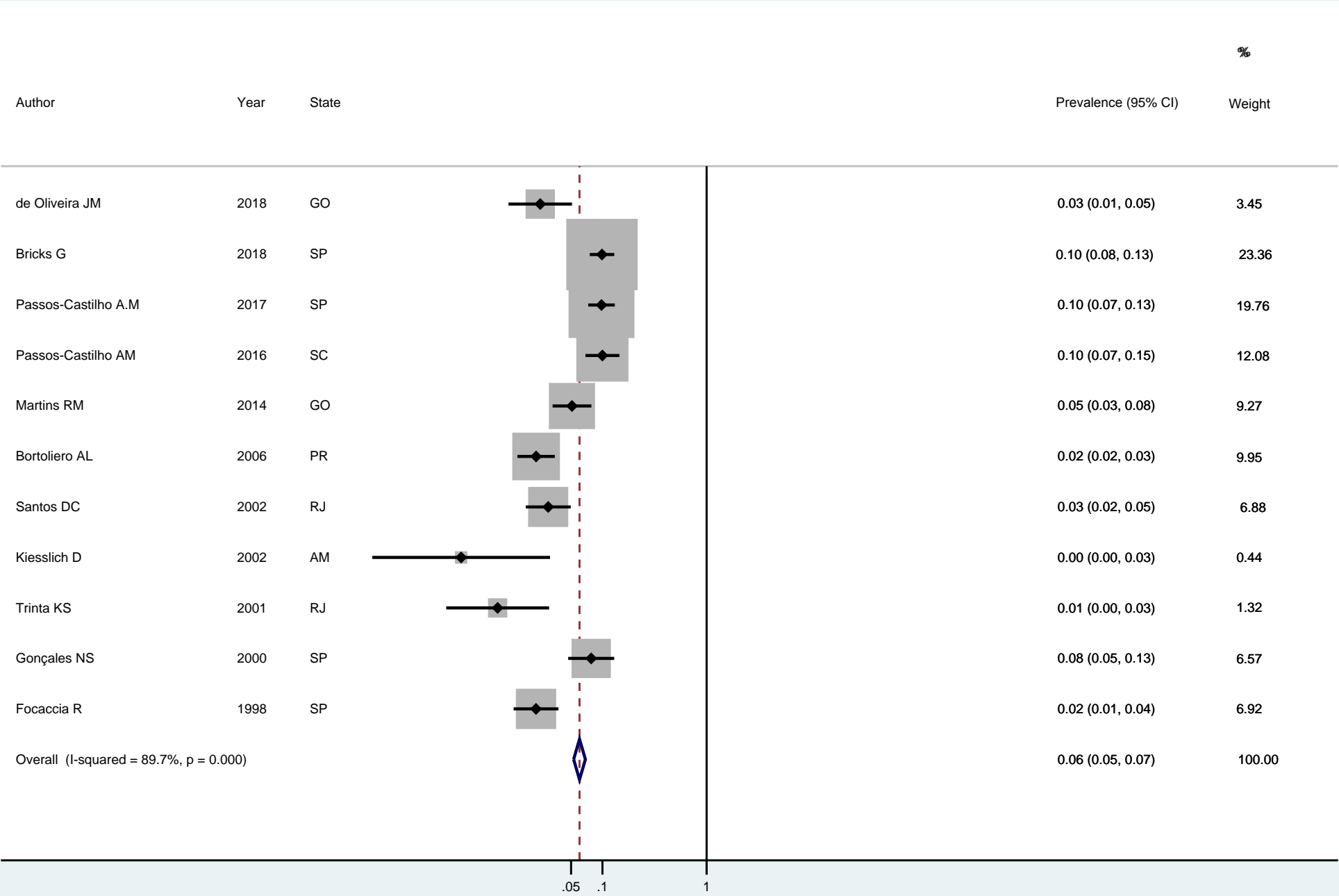

Prevalence

Supplement: Supplementary file 8 — Seroprevalence of hepatitis E in studies with a sample size > 200. (PDF 26 kb) [file 40249_2018_514_MOESM8_ESM.pdf]
