# Supplementary material for: Seroprevalence of hepatitis E in adults in Brazil: a systematic review and meta-analysis
Source: Infect Dis Poverty. 2019 Jan 16;8:3. doi: 10.1186/s40249-018-0514-4 (PMC6334402; doi:10.1186/s40249-018-0514-4)
Supplement: Supplementary file 5 — Tests for Publication Bias. Tests for Publication Bias. (DOCX 11 kb) [file 40249_2018_514_MOESM5_ESM.docx]

. metabias logo selogo, graph (begg)

Note: default data input format (theta, se_theta) assumed.

**Tests for Publication Bias**

**Begg's Test**

adj. Kendall's Score (P-Q) = -41

Std. Dev. of Score = 26.40

Number of Studies = 18

|z| = -1.55

Pr > |z| = 0.120

|z| = 1.52 (continuity corrected)

Pr > |z| = 0.130 (continuity corrected)

**Egger's test**

Std_Eff Coef. Std. Err. t P>t [95% Conf. Interval]

slope -2.073972 .263645 -7.87 0.000 -2.632874 -1.51507

bias -3.32034 .9876558 -3.36 0.004 -5.414076 -1.226603

.
